# 4-Methyl-*N*-(4-methyl­phenyl­sulfon­yl)-*N*-[4-(4-methyl­phen­yl)-1,3-thia­zol-2-yl]benzene­sulfonamide

**DOI:** 10.1107/S1600536813032145

**Published:** 2013-12-04

**Authors:** Rubén M. Carballo, Simón Hernández-Ortega, Nayely Padilla-Montaño, Reyna Reyes-Martínez, Gumersindo Mirón-López

**Affiliations:** a Facultad de Química, Universidad, Autónoma de Yucatán, Calle 41 No. 421, Col. Industrial, CP 97150, Mérida, Yucatán, Mexico; b Instituto de Química, Universidad Nacional Autónoma de México, Circuito exterior, Ciudad Universitaria, México, DF 04510, Mexico

## Abstract

There are two independent mol­ecules in the asymmetric unit of the title compound, C_24_H_22_N_2_O_4_S_3_. In each, the sulfonamide N atoms reveal nearly a trigonal-planar geometry with two S atoms of the O=S=O groups and one C atom of the thia­zole ring; the angles around the N atoms are between 117.00 (13) and 123.86 (9)°. The methyl­phenyl­sulfonyl groups are in *anti* conformations, forming dihedral angles of 78.00 (7)/72.53 (5) and 77.09 (6)/71.50 (7)° with the trigonal S—N—S planes in the two mol­ecules. The thia­zole groups are rotated around the C—N bonds and are almost perpendicular to the S—N—S plane [dihedral angles of 78.00 (7)/72.53 (5) and 77.09 (6)/71.50 (7)°]. In the crystal, pairs of C—H⋯O inter­actions, with the O atoms of the sulfonamide groups as acceptors, link each of the independent mol­ecules into inversion dimers.

## Related literature   

For bioactive sulfonamide compounds, see: Annadurai *et al.* (2012 ▶); Farag *et al.* (2012 ▶); Xiao-Long *et al.* (2009 ▶).
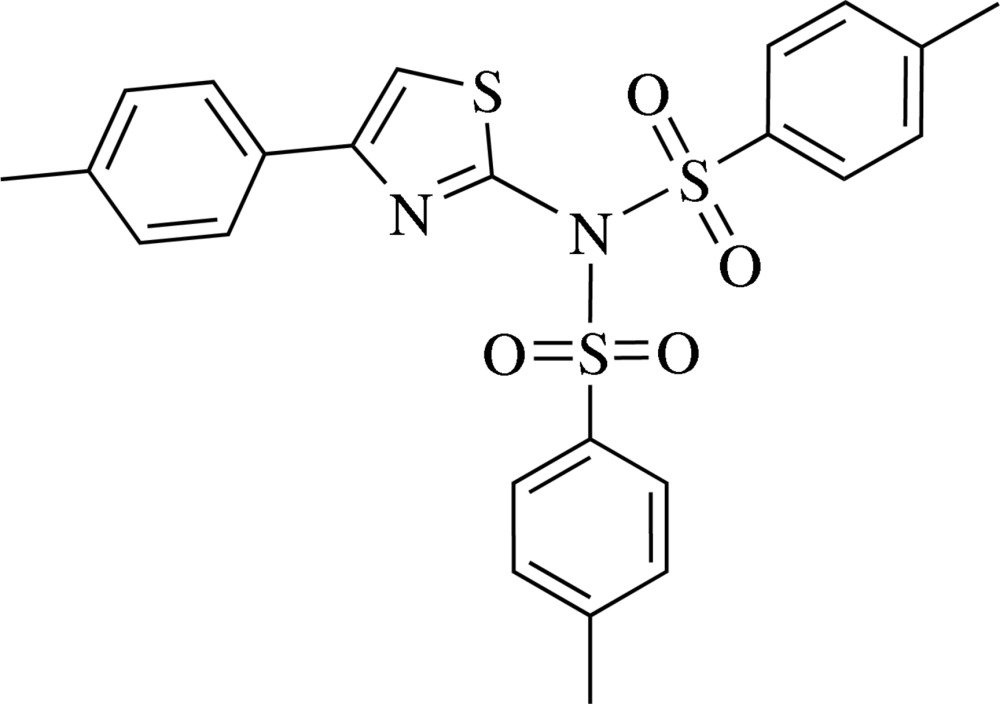


## Experimental   

### 

#### Crystal data  

C_24_H_22_N_2_O_4_S_3_
*M* *_r_* = 498.61
Triclinic, 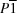

*a* = 8.3322 (2) Å
*b* = 12.0630 (3) Å
*c* = 23.5756 (6) Å
α = 84.615 (1)°
β = 87.022 (1)°
γ = 85.482 (1)°
*V* = 2349.46 (10) Å^3^
*Z* = 4
Mo *K*α radiation
μ = 0.35 mm^−1^
*T* = 298 K
0.44 × 0.38 × 0.28 mm

#### Data collection  

Bruker APEXII CCD area-detector diffractometer
18770 measured reflections
8539 independent reflections
6755 reflections with *I* > 2σ(*I*)
*R*_int_ = 0.035

#### Refinement  

*R*[*F* ^2^ > 2σ(*F* ^2^)] = 0.039
*wR*(*F* ^2^) = 0.104
*S* = 0.98
8539 reflections
602 parameters
2 restraints
H-atom parameters constrained
Δρ_max_ = 0.23 e Å^−3^
Δρ_min_ = −0.28 e Å^−3^

### 

Data collection: *APEX2* (Bruker, 2007 ▶); cell refinement: *SAINT* (Bruker, 2007 ▶); data reduction: *SAINT*; program(s) used to solve structure: *SHELXS97* (Sheldrick, 2008 ▶); program(s) used to refine structure: *SHELXL2013* (Sheldrick, 2008 ▶); molecular graphics: *ORTEP-3 for Windows* (Farrugia, 2012 ▶) and *DIAMOND* (Brandenburg, 2006 ▶); software used to prepare material for publication: *SHELXTL* (Sheldrick, 2008 ▶) and *PLATON* (Spek, 2009 ▶).

## Supplementary Material

Crystal structure: contains datablock(s) I, global. DOI: 10.1107/S1600536813032145/kp2458sup1.cif

Structure factors: contains datablock(s) I. DOI: 10.1107/S1600536813032145/kp2458Isup2.hkl

Additional supporting information:  crystallographic information; 3D view; checkCIF report

## Figures and Tables

**Table 1:** Hydrogen-bond geometry (Å, °)

*D*—H⋯*A*	*D*—H	H⋯*A*	*D*⋯*A*	*D*—H⋯*A*
C29—H29*B*⋯O3^i^	0.96	2.54	3.148 (3)	122
C50—H50*C*⋯O5^ii^	0.96	2.47	3.397 (3)	162

Symmetry codes: (i) 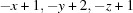; (ii) 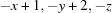.

**Fig. 1.**
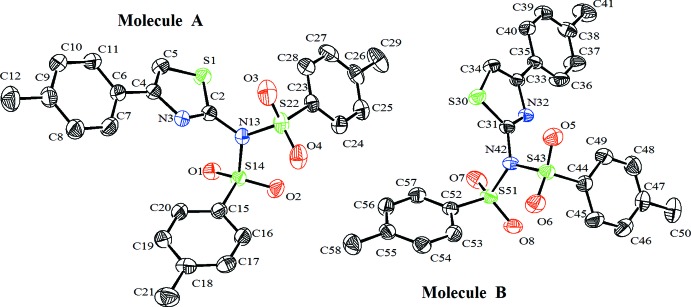
The asymmetric unit of the title compound (I). All non-hydrogen atoms are shown as ellipsoids with probability level of 40%. Hydrogen atoms are omitted.

**Fig. 2.**
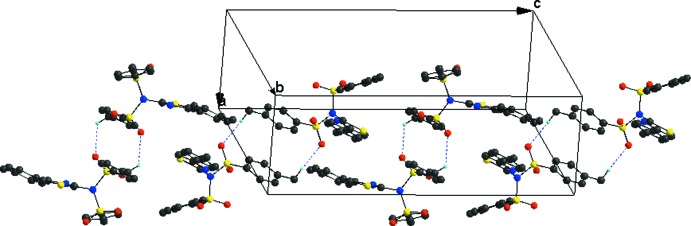
Hydrogen bonds in the crystal packing of the title compound, shown by dashed lines.

## supplementary crystallographic information

### 1. Comment

Sulfonamide thiazoles are structural units frequently found as parts of skeletons of bioactive compounds including antimicrobials agents (Annadurai *et al.*, 2012), anticonvulsant agents (Farag *et al.*, 2012) or inhibitors of Nek2/Hec1 (Xiao-Long *et al.*, 2009). Due to the importance of thiazole derivatives, we synthesized (I) *N*-[4-(*p*-tolyl)thiazol-2-yl]-4-methyl-*N*-(4- methylphenylsulfonyl)benzenesulfonamide, and discuss the geometry of the molecule and its conformation. The compound was obtained by the reaction of 4-*p*-tolyl-thiazol-2-ylamine in an excess of 4-methyl-benzenesulfonilchloride.

The title compound I crystallized with two independent molecules (A and B) in the asymmetric unit (Fig. 1). The geometries around the N13 and N42 atoms are almost trigonal planar with bonding angles ranging from 117.00° to 123.86°. The sulfonamide nitrogen atoms are bonded to a carbon atom of the thiazole ring and two sulfur atoms of the O=S=O groups, the distances of the C—N bonds are of 1.426 (2) Å for both molecules, and the N—S bonds are between 1.6902 (16) and 1.7040 (17) Å. The thiazole rings are rotated around C—N bond foming the dihedral angles of 85.57 (5)° and 89.28 (5)° with the planes O2S—N—SO2, in molecule A and B, respectively. The methylphenylsulfonyl groups keep anti-conformations forming a dihedral angles of 78.00 (7)°, 72.53 (5)° and 77.09 (6)°, 71.50 (7)° with the trigonal plane S—N—S, in the molecules A and B, respectively. The sulfur atoms of the sulfonamide groups are in a distorted tetrahedral geometry with angles varying from 103.43 (8) to 121.39 (1)°, and S=O bonds from 1.4175 (15) to 1.42358 (14) Å. The thiazole and *p*-tolyl groups in the molecule B exhibit a coplanar arrangement, while in the molecule A these moieties form an angle of 17.20 (7)° between these planes. Each independent molecule form a dimer arrangement by C—H···O interactions (Table 1, Fig. 2). The crystal packing in the title compound is stabilized by the C—H···O=S intermolecular interactions.

### 2. Experimental

A mixture of 4-*p*-tolyl-thiazol-2-ylamine (200 mg, 1.05 mmol), 4-methyl-benzenesulfonyl chloride (600 mg, 3.15 mmol), triethyl-amine (0.293 mL, 2.1 mmol) and dimethyl-pyridin-4-yl-amine (12.8 mg, 0.105 mmol) in dichloromethane (0.1 *M*, 11 mL) was stirred in a 50 mL round bottom flask at room temperature for 12 h, and the reaction was monitored by TLC. Then, the reaction solution was quenched by addition of water with stirring and extracted with dichloromethane. The organic layers were dried over magnesium sulfate, and the solvent was removed under reduced pressure. The residue was crystallized from acetone to yield single crystals of the title compound (314 mg, 0.63 mmol, 60%).

### 3. Refinement

H atoms were included in calculated position (C—H = 0.93 Å for aromatic H, and C—H = 0.96 Å for methyl H), and refined using a riding model *U_iso_*(H) = 1.2 *U_eq_* of the carrier atoms. In the refinement 10 reflections were considered as disagreeable and were omitted.

### Crystal data

**Table:**

C_24_H_22_N_2_O_4_S_3_	*Z* = 4
*M**_r_* = 498.61	*F*(000) = 1040
Triclinic, *P*1	*D*_x_ = 1.410 Mg m^−^^3^
*a* = 8.3322 (2) Å	Mo *K*α radiation, λ = 0.71073 Å
*b* = 12.0630 (3) Å	Cell parameters from 7195 reflections
*c* = 23.5756 (6) Å	θ = 2.3–25.3°
α = 84.615 (1)°	µ = 0.35 mm^−^^1^
β = 87.022 (1)°	*T* = 298 K
γ = 85.482 (1)°	Prism, colourless
*V* = 2349.46 (10) Å^3^	0.44 × 0.38 × 0.28 mm

### Data collection

**Table:**

Bruker APEXII CCD area-detector diffractometer	*R*_int_ = 0.035
Detector resolution: 0.83 pixels mm^-1^	θ_max_ = 25.4°, θ_min_ = 1.7°
ω scans	*h* = −10→10
18770 measured reflections	*k* = −14→14
8539 independent reflections	*l* = −28→28
6755 reflections with *I* > 2σ(*I*)	

### Refinement

**Table:**

Refinement on *F*^2^	Hydrogen site location: inferred from neighbouring sites
Least-squares matrix: full	H-atom parameters constrained
*R*[*F*^2^ > 2σ(*F*^2^)] = 0.039	*w* = 1/[σ^2^(*F*_o_^2^) + (0.0613*P*)^2^]
*wR*(*F*^2^) = 0.104	(Δ/σ)_max_ = 0.002
*S* = 0.98	Δρ_max_ = 0.23 e Å^−^^3^
8539 reflections	Δρ_min_ = −0.28 e Å^−^^3^
602 parameters	Extinction correction: *SHELXL2013* (Sheldrick, 2008), Fc^*^=kFc[1+0.001xFc^2^λ^3^/sin(2θ)]^-1/4^
2 restraints	Extinction coefficient: 0.0092 (7)

### Special details

**Table:**

Geometry. All e.s.d.'s (except the e.s.d. in the dihedral angle between two l.s. planes) are estimated using the full covariance matrix. The cell e.s.d.'s are taken into account individually in the estimation of e.s.d.'s in distances, angles and torsion angles; correlations between e.s.d.'s in cell parameters are only used when they are defined by crystal symmetry. An approximate (isotropic) treatment of cell e.s.d.'s is used for estimating e.s.d.'s involving l.s. planes.

### Fractional atomic coordinates and isotropic or equivalent isotropic displacement parameters (Å^2^)

**Table:**

	*x*	*y*	*z*	*U*_iso_*/*U*_eq_	
S1	0.21107 (6)	0.86099 (4)	0.70159 (2)	0.04992 (16)	
C2	0.3027 (2)	0.73811 (15)	0.68033 (8)	0.0403 (4)	
N3	0.38103 (19)	0.67554 (13)	0.71866 (7)	0.0418 (4)	
C4	0.3706 (2)	0.72444 (16)	0.76934 (8)	0.0411 (5)	
C5	0.2846 (2)	0.82565 (16)	0.76730 (9)	0.0473 (5)	
H5	0.2683	0.8695	0.7978	0.057*	
C6	0.4503 (2)	0.66461 (16)	0.81880 (8)	0.0424 (5)	
C7	0.4960 (3)	0.55130 (18)	0.81897 (9)	0.0555 (6)	
H7	0.4752	0.5137	0.7878	0.067*	
C8	0.5717 (3)	0.49367 (18)	0.86461 (9)	0.0585 (6)	
H8	0.6000	0.4177	0.8638	0.070*	
C9	0.6060 (3)	0.54611 (19)	0.91126 (9)	0.0532 (5)	
C10	0.5589 (3)	0.65886 (18)	0.91154 (9)	0.0607 (6)	
H10	0.5792	0.6959	0.9430	0.073*	
C11	0.4825 (3)	0.71747 (17)	0.86615 (9)	0.0546 (6)	
H11	0.4524	0.7931	0.8674	0.066*	
C12	0.6947 (3)	0.4832 (2)	0.96038 (10)	0.0776 (8)	
H12A	0.7016	0.5316	0.9901	0.116*	
H12B	0.8013	0.4583	0.9472	0.116*	
H12C	0.6374	0.4198	0.9749	0.116*	
N13	0.29416 (19)	0.70889 (13)	0.62341 (6)	0.0424 (4)	
S14	0.15899 (6)	0.61754 (4)	0.61109 (2)	0.04159 (14)	
O1	0.02760 (15)	0.64008 (11)	0.65010 (6)	0.0485 (3)	
O2	0.13878 (17)	0.63097 (12)	0.55121 (6)	0.0551 (4)	
C15	0.2485 (2)	0.48402 (16)	0.63075 (8)	0.0418 (5)	
C16	0.3208 (2)	0.41966 (17)	0.58949 (9)	0.0495 (5)	
H16	0.3258	0.4477	0.5514	0.059*	
C17	0.3850 (2)	0.31386 (17)	0.60579 (9)	0.0524 (5)	
H17	0.4324	0.2701	0.5781	0.063*	
C18	0.3811 (2)	0.27080 (16)	0.66211 (10)	0.0503 (5)	
C19	0.3084 (3)	0.33669 (17)	0.70260 (9)	0.0537 (6)	
H19	0.3047	0.3087	0.7407	0.064*	
C20	0.2412 (3)	0.44314 (16)	0.68760 (8)	0.0492 (5)	
H20	0.1920	0.4864	0.7152	0.059*	
C21	0.4539 (3)	0.15517 (18)	0.67975 (12)	0.0743 (7)	
H21A	0.5487	0.1604	0.7005	0.112*	
H21B	0.4823	0.1166	0.6464	0.112*	
H21C	0.3770	0.1148	0.7035	0.112*	
S22	0.44065 (6)	0.75042 (5)	0.57530 (2)	0.04896 (16)	
O3	0.55605 (16)	0.79107 (15)	0.60897 (7)	0.0711 (5)	
O4	0.48252 (19)	0.66221 (13)	0.54054 (6)	0.0672 (5)	
C23	0.3487 (2)	0.86359 (17)	0.53395 (8)	0.0429 (5)	
C24	0.2862 (2)	0.84817 (18)	0.48251 (8)	0.0488 (5)	
H24	0.2953	0.7777	0.4692	0.059*	
C25	0.2106 (3)	0.93665 (16)	0.45099 (9)	0.0551 (6)	
H25	0.1711	0.9257	0.4159	0.066*	
C26	0.1920 (2)	1.04194 (16)	0.47036 (9)	0.0550 (6)	
C27	0.2570 (3)	1.05608 (18)	0.52176 (9)	0.0642 (7)	
H27	0.2477	1.1265	0.5351	0.077*	
C28	0.3354 (3)	0.96839 (18)	0.55381 (10)	0.0572 (6)	
H28	0.3787	0.9797	0.5882	0.069*	
C29	0.1023 (3)	1.1370 (2)	0.43677 (12)	0.0802 (8)	
H29A	−0.0054	1.1181	0.4317	0.120*	
H29B	0.1566	1.1511	0.4002	0.120*	
H29C	0.0986	1.2027	0.4570	0.120*	
S30	0.28190 (7)	1.08557 (4)	0.27908 (2)	0.05011 (16)	
C31	0.2102 (2)	1.04886 (15)	0.21732 (8)	0.0399 (4)	
N32	0.13792 (19)	1.12839 (12)	0.18516 (7)	0.0408 (4)	
C33	0.1363 (2)	1.22799 (14)	0.21026 (8)	0.0391 (4)	
C34	0.2082 (2)	1.21888 (15)	0.26092 (9)	0.0456 (5)	
H34	0.2162	1.2781	0.2830	0.055*	
C35	0.0581 (2)	1.32933 (15)	0.18075 (8)	0.0401 (4)	
C36	−0.0114 (3)	1.32644 (17)	0.12923 (10)	0.0622 (6)	
H36	−0.0078	1.2594	0.1125	0.075*	
C37	−0.0862 (3)	1.42155 (18)	0.10196 (11)	0.0737 (8)	
H37	−0.1341	1.4168	0.0676	0.088*	
C38	−0.0918 (3)	1.52323 (17)	0.12436 (11)	0.0576 (6)	
C39	−0.0221 (3)	1.52557 (17)	0.17562 (10)	0.0586 (6)	
H39	−0.0244	1.5929	0.1920	0.070*	
C40	0.0512 (3)	1.43090 (15)	0.20370 (9)	0.0513 (5)	
H40	0.0965	1.4356	0.2385	0.062*	
C41	−0.1697 (3)	1.62846 (19)	0.09396 (13)	0.0871 (9)	
H41A	−0.1077	1.6484	0.0596	0.131*	
H41B	−0.1738	1.6881	0.1184	0.131*	
H41C	−0.2771	1.6156	0.0847	0.131*	
N42	0.23294 (19)	0.93682 (12)	0.20213 (7)	0.0427 (4)	
S43	0.39249 (6)	0.90607 (4)	0.15665 (2)	0.04773 (15)	
O5	0.50268 (17)	0.98571 (12)	0.16599 (7)	0.0583 (4)	
O6	0.43414 (19)	0.78969 (11)	0.16812 (6)	0.0624 (4)	
C44	0.3245 (3)	0.93295 (16)	0.08724 (9)	0.0495 (5)	
C45	0.2867 (3)	0.84603 (19)	0.05707 (10)	0.0693 (7)	
H45	0.2966	0.7729	0.0736	0.083*	
C46	0.2344 (3)	0.8686 (2)	0.00260 (10)	0.0729 (7)	
H46	0.2102	0.8099	−0.0176	0.088*	
C47	0.2170 (3)	0.9760 (2)	−0.02279 (9)	0.0597 (6)	
C48	0.2570 (3)	1.0612 (2)	0.00777 (10)	0.0663 (7)	
H48	0.2473	1.1342	−0.0089	0.080*	
C49	0.3109 (3)	1.04112 (18)	0.06221 (10)	0.0586 (6)	
H49	0.3378	1.0998	0.0819	0.070*	
C50	0.1540 (3)	1.0004 (2)	−0.08219 (10)	0.0806 (8)	
H50A	0.0718	1.0608	−0.0823	0.121*	
H50B	0.1096	0.9350	−0.0933	0.121*	
H50C	0.2407	1.0207	−0.1086	0.121*	
S51	0.07665 (6)	0.85425 (4)	0.21475 (2)	0.04588 (15)	
O7	−0.05860 (17)	0.92715 (11)	0.22956 (7)	0.0609 (4)	
O8	0.0765 (2)	0.79145 (11)	0.16686 (6)	0.0617 (4)	
C52	0.1246 (2)	0.76319 (15)	0.27528 (8)	0.0412 (5)	
C53	0.2258 (3)	0.66786 (16)	0.26941 (9)	0.0521 (5)	
H53	0.2736	0.6537	0.2340	0.063*	
C54	0.2545 (3)	0.59416 (17)	0.31684 (9)	0.0527 (5)	
H54	0.3225	0.5301	0.3130	0.063*	
C55	0.1849 (2)	0.61315 (17)	0.36979 (9)	0.0473 (5)	
C56	0.0841 (3)	0.70896 (18)	0.37416 (9)	0.0544 (6)	
H56	0.0354	0.7230	0.4094	0.065*	
C57	0.0541 (2)	0.78400 (17)	0.32762 (9)	0.0492 (5)	
H57	−0.0134	0.8483	0.3315	0.059*	
C58	0.2167 (3)	0.53202 (19)	0.42073 (10)	0.0661 (7)	
H58A	0.3187	0.5439	0.4354	0.099*	
H58B	0.2187	0.4572	0.4099	0.099*	
H58C	0.1329	0.5430	0.4496	0.099*	

### Atomic displacement parameters (Å^2^)

**Table:**

	*U*^11^	*U*^22^	*U*^33^	*U*^12^	*U*^13^	*U*^23^
S1	0.0562 (3)	0.0432 (3)	0.0499 (3)	0.0036 (3)	−0.0051 (3)	−0.0061 (2)
C2	0.0424 (11)	0.0394 (11)	0.0395 (11)	−0.0056 (9)	0.0001 (9)	−0.0044 (9)
N3	0.0496 (10)	0.0396 (9)	0.0364 (9)	−0.0057 (8)	−0.0008 (7)	−0.0035 (7)
C4	0.0433 (11)	0.0419 (11)	0.0390 (11)	−0.0090 (9)	0.0011 (9)	−0.0053 (9)
C5	0.0532 (12)	0.0457 (12)	0.0444 (12)	−0.0041 (10)	−0.0011 (10)	−0.0111 (10)
C6	0.0462 (11)	0.0444 (11)	0.0373 (11)	−0.0095 (9)	0.0018 (9)	−0.0035 (9)
C7	0.0742 (15)	0.0514 (13)	0.0414 (12)	0.0026 (11)	−0.0050 (11)	−0.0116 (10)
C8	0.0770 (16)	0.0492 (13)	0.0474 (13)	0.0061 (12)	−0.0031 (12)	−0.0032 (11)
C9	0.0587 (13)	0.0574 (14)	0.0423 (12)	−0.0075 (11)	−0.0011 (10)	0.0047 (10)
C10	0.0870 (17)	0.0552 (14)	0.0429 (12)	−0.0159 (13)	−0.0137 (12)	−0.0050 (11)
C11	0.0755 (15)	0.0415 (12)	0.0486 (13)	−0.0082 (11)	−0.0107 (11)	−0.0058 (10)
C12	0.098 (2)	0.0774 (18)	0.0538 (15)	0.0031 (15)	−0.0145 (14)	0.0104 (13)
N13	0.0477 (9)	0.0453 (9)	0.0341 (9)	−0.0068 (8)	0.0001 (7)	−0.0011 (7)
S14	0.0445 (3)	0.0420 (3)	0.0381 (3)	0.0013 (2)	−0.0042 (2)	−0.0050 (2)
O1	0.0406 (7)	0.0500 (8)	0.0549 (9)	−0.0001 (6)	0.0021 (6)	−0.0089 (7)
O2	0.0681 (10)	0.0569 (9)	0.0405 (8)	0.0041 (7)	−0.0144 (7)	−0.0059 (7)
C15	0.0442 (11)	0.0406 (11)	0.0409 (11)	−0.0020 (9)	−0.0034 (9)	−0.0048 (9)
C16	0.0550 (13)	0.0512 (13)	0.0421 (12)	0.0001 (10)	0.0004 (10)	−0.0078 (10)
C17	0.0543 (13)	0.0478 (13)	0.0557 (14)	0.0035 (10)	0.0012 (11)	−0.0161 (11)
C18	0.0491 (12)	0.0396 (11)	0.0636 (14)	−0.0020 (10)	−0.0117 (11)	−0.0085 (11)
C19	0.0700 (14)	0.0450 (12)	0.0463 (12)	−0.0052 (11)	−0.0106 (11)	0.0007 (10)
C20	0.0624 (13)	0.0454 (12)	0.0398 (11)	−0.0003 (10)	−0.0012 (10)	−0.0079 (10)
C21	0.0869 (18)	0.0474 (14)	0.0889 (19)	0.0057 (13)	−0.0210 (15)	−0.0068 (13)
S22	0.0385 (3)	0.0630 (4)	0.0421 (3)	0.0029 (3)	0.0012 (2)	0.0052 (3)
O3	0.0409 (8)	0.1111 (14)	0.0605 (10)	−0.0190 (9)	−0.0101 (7)	0.0124 (9)
O4	0.0708 (10)	0.0706 (10)	0.0521 (9)	0.0262 (9)	0.0135 (8)	0.0011 (8)
C23	0.0355 (10)	0.0507 (12)	0.0410 (11)	−0.0065 (9)	0.0047 (9)	0.0023 (9)
C24	0.0484 (12)	0.0533 (13)	0.0439 (12)	−0.0022 (10)	0.0021 (10)	−0.0033 (10)
C25	0.0506 (13)	0.0637 (15)	0.0484 (13)	−0.0023 (11)	−0.0023 (10)	0.0076 (11)
C26	0.0393 (11)	0.0575 (14)	0.0633 (15)	−0.0044 (10)	0.0119 (11)	0.0126 (12)
C27	0.0676 (15)	0.0415 (13)	0.0817 (18)	−0.0110 (11)	0.0177 (14)	−0.0013 (12)
C28	0.0638 (14)	0.0551 (14)	0.0543 (14)	−0.0183 (11)	0.0014 (11)	−0.0040 (11)
C29	0.0538 (15)	0.0697 (16)	0.106 (2)	0.0090 (13)	0.0105 (14)	0.0330 (16)
S30	0.0628 (3)	0.0406 (3)	0.0472 (3)	0.0031 (3)	−0.0096 (3)	−0.0078 (2)
C31	0.0448 (11)	0.0307 (10)	0.0441 (11)	−0.0025 (8)	0.0014 (9)	−0.0048 (9)
N32	0.0477 (9)	0.0303 (8)	0.0447 (9)	−0.0046 (7)	0.0005 (8)	−0.0045 (7)
C33	0.0418 (11)	0.0294 (10)	0.0464 (11)	−0.0056 (8)	0.0058 (9)	−0.0064 (8)
C34	0.0533 (12)	0.0352 (11)	0.0499 (12)	−0.0016 (9)	−0.0021 (10)	−0.0138 (9)
C35	0.0414 (11)	0.0305 (10)	0.0481 (12)	−0.0048 (8)	0.0038 (9)	−0.0037 (9)
C36	0.0848 (17)	0.0341 (11)	0.0700 (16)	−0.0015 (11)	−0.0209 (13)	−0.0089 (11)
C37	0.099 (2)	0.0474 (14)	0.0774 (17)	−0.0050 (13)	−0.0406 (16)	−0.0010 (13)
C38	0.0573 (14)	0.0391 (12)	0.0754 (16)	−0.0032 (10)	−0.0086 (12)	0.0024 (11)
C39	0.0761 (16)	0.0300 (11)	0.0693 (16)	−0.0033 (11)	0.0016 (13)	−0.0060 (11)
C40	0.0676 (14)	0.0357 (11)	0.0506 (13)	−0.0023 (10)	−0.0015 (11)	−0.0063 (10)
C41	0.101 (2)	0.0458 (14)	0.113 (2)	0.0006 (14)	−0.0331 (19)	0.0110 (15)
N42	0.0532 (10)	0.0278 (8)	0.0475 (10)	−0.0025 (7)	−0.0005 (8)	−0.0058 (7)
S43	0.0545 (3)	0.0376 (3)	0.0503 (3)	0.0057 (2)	−0.0025 (2)	−0.0075 (2)
O5	0.0498 (9)	0.0597 (9)	0.0670 (10)	−0.0061 (7)	−0.0005 (7)	−0.0143 (8)
O6	0.0797 (11)	0.0424 (8)	0.0624 (10)	0.0193 (8)	−0.0059 (8)	−0.0084 (7)
C44	0.0606 (13)	0.0402 (11)	0.0468 (12)	0.0015 (10)	0.0025 (10)	−0.0060 (10)
C45	0.110 (2)	0.0426 (13)	0.0558 (15)	−0.0027 (13)	−0.0055 (14)	−0.0105 (11)
C46	0.104 (2)	0.0657 (16)	0.0516 (15)	−0.0078 (15)	−0.0056 (14)	−0.0164 (13)
C47	0.0567 (14)	0.0750 (16)	0.0455 (13)	−0.0018 (12)	0.0096 (11)	−0.0046 (12)
C48	0.0803 (17)	0.0568 (14)	0.0582 (15)	−0.0015 (13)	0.0010 (13)	0.0077 (12)
C49	0.0764 (16)	0.0436 (12)	0.0558 (14)	−0.0073 (11)	−0.0022 (12)	−0.0030 (11)
C50	0.0746 (17)	0.114 (2)	0.0497 (15)	−0.0005 (16)	0.0013 (13)	0.0010 (15)
S51	0.0541 (3)	0.0318 (3)	0.0524 (3)	−0.0050 (2)	−0.0133 (3)	0.0005 (2)
O7	0.0466 (8)	0.0460 (8)	0.0875 (12)	0.0033 (7)	−0.0084 (8)	0.0056 (8)
O8	0.0960 (12)	0.0406 (8)	0.0521 (9)	−0.0172 (8)	−0.0256 (8)	−0.0001 (7)
C52	0.0459 (11)	0.0328 (10)	0.0462 (11)	−0.0053 (9)	−0.0095 (9)	−0.0034 (9)
C53	0.0693 (14)	0.0417 (12)	0.0439 (12)	0.0028 (11)	−0.0012 (11)	−0.0024 (10)
C54	0.0622 (14)	0.0393 (11)	0.0555 (14)	0.0046 (10)	−0.0086 (11)	−0.0016 (10)
C55	0.0506 (12)	0.0477 (12)	0.0455 (12)	−0.0141 (10)	−0.0114 (10)	−0.0005 (10)
C56	0.0582 (13)	0.0598 (14)	0.0459 (12)	−0.0090 (11)	0.0022 (10)	−0.0063 (11)
C57	0.0492 (12)	0.0433 (12)	0.0553 (13)	−0.0007 (10)	−0.0015 (10)	−0.0086 (10)
C58	0.0751 (16)	0.0686 (15)	0.0541 (14)	−0.0109 (13)	−0.0175 (12)	0.0095 (12)

### Geometric parameters (Å, º)

**Table:**

S1—C5	1.700 (2)	S30—C34	1.6998 (19)
S1—C2	1.7182 (19)	S30—C31	1.7107 (19)
C2—N3	1.292 (2)	C31—N32	1.295 (2)
C2—N13	1.426 (2)	C31—N42	1.426 (2)
N3—C4	1.377 (2)	N32—C33	1.387 (2)
C4—C5	1.364 (3)	C33—C34	1.355 (3)
C4—C6	1.473 (3)	C33—C35	1.471 (2)
C5—H5	0.9300	C34—H34	0.9300
C6—C11	1.384 (3)	C35—C36	1.377 (3)
C6—C7	1.389 (3)	C35—C40	1.381 (3)
C7—C8	1.378 (3)	C36—C37	1.382 (3)
C7—H7	0.9300	C36—H36	0.9300
C8—C9	1.372 (3)	C37—C38	1.377 (3)
C8—H8	0.9300	C37—H37	0.9300
C9—C10	1.386 (3)	C38—C39	1.371 (3)
C9—C12	1.516 (3)	C38—C41	1.515 (3)
C10—C11	1.381 (3)	C39—C40	1.380 (3)
C10—H10	0.9300	C39—H39	0.9300
C11—H11	0.9300	C40—H40	0.9300
C12—H12A	0.9600	C41—H41A	0.9600
C12—H12B	0.9600	C41—H41B	0.9600
C12—H12C	0.9600	C41—H41C	0.9600
N13—S14	1.6902 (16)	N42—S51	1.6985 (17)
N13—S22	1.6951 (16)	N42—S43	1.7040 (17)
S14—O1	1.4190 (14)	S43—O5	1.4191 (15)
S14—O2	1.4232 (14)	S43—O6	1.4235 (14)
S14—C15	1.7526 (19)	S43—C44	1.752 (2)
C15—C20	1.384 (3)	C44—C49	1.380 (3)
C15—C16	1.386 (3)	C44—C45	1.386 (3)
C16—C17	1.373 (3)	C45—C46	1.374 (3)
C16—H16	0.9300	C45—H45	0.9300
C17—C18	1.379 (3)	C46—C47	1.375 (3)
C17—H17	0.9300	C46—H46	0.9300
C18—C19	1.384 (3)	C47—C48	1.379 (3)
C18—C21	1.507 (3)	C47—C50	1.516 (3)
C19—C20	1.382 (3)	C48—C49	1.375 (3)
C19—H19	0.9300	C48—H48	0.9300
C20—H20	0.9300	C49—H49	0.9300
C21—H21A	0.9600	C50—H50A	0.9600
C21—H21B	0.9600	C50—H50B	0.9600
C21—H21C	0.9600	C50—H50C	0.9600
S22—O4	1.4146 (16)	S51—O8	1.4175 (15)
S22—O3	1.4219 (15)	S51—O7	1.4233 (15)
S22—C23	1.7505 (19)	S51—C52	1.7605 (19)
C23—C24	1.378 (3)	C52—C57	1.375 (3)
C23—C28	1.383 (3)	C52—C53	1.384 (3)
C24—C25	1.372 (3)	C53—C54	1.380 (3)
C24—H24	0.9300	C53—H53	0.9300
C25—C26	1.3842 (17)	C54—C55	1.380 (3)
C25—H25	0.9300	C54—H54	0.9300
C26—C27	1.3831 (17)	C55—C56	1.383 (3)
C26—C29	1.502 (3)	C55—C58	1.497 (3)
C27—C28	1.382 (3)	C56—C57	1.376 (3)
C27—H27	0.9300	C56—H56	0.9300
C28—H28	0.9300	C57—H57	0.9300
C29—H29A	0.9600	C58—H58A	0.9600
C29—H29B	0.9600	C58—H58B	0.9600
C29—H29C	0.9600	C58—H58C	0.9600
			
C5—S1—C2	88.51 (9)	C34—S30—C31	88.39 (9)
N3—C2—N13	122.53 (17)	N32—C31—N42	122.52 (17)
N3—C2—S1	115.95 (14)	N32—C31—S30	116.37 (14)
N13—C2—S1	121.50 (14)	N42—C31—S30	121.10 (14)
C2—N3—C4	110.23 (16)	C31—N32—C33	109.64 (16)
C5—C4—N3	114.31 (17)	C34—C33—N32	114.17 (16)
C5—C4—C6	127.27 (18)	C34—C33—C35	127.37 (17)
N3—C4—C6	118.42 (16)	N32—C33—C35	118.45 (17)
C4—C5—S1	110.99 (15)	C33—C34—S30	111.43 (14)
C4—C5—H5	124.5	C33—C34—H34	124.3
S1—C5—H5	124.5	S30—C34—H34	124.3
C11—C6—C7	117.75 (18)	C36—C35—C40	117.44 (18)
C11—C6—C4	122.21 (18)	C36—C35—C33	121.25 (17)
C7—C6—C4	120.04 (17)	C40—C35—C33	121.31 (18)
C8—C7—C6	121.09 (19)	C35—C36—C37	121.1 (2)
C8—C7—H7	119.5	C35—C36—H36	119.5
C6—C7—H7	119.5	C37—C36—H36	119.5
C9—C8—C7	121.3 (2)	C38—C37—C36	121.7 (2)
C9—C8—H8	119.3	C38—C37—H37	119.2
C7—C8—H8	119.3	C36—C37—H37	119.2
C8—C9—C10	117.7 (2)	C39—C38—C37	116.9 (2)
C8—C9—C12	121.1 (2)	C39—C38—C41	121.0 (2)
C10—C9—C12	121.2 (2)	C37—C38—C41	122.0 (2)
C11—C10—C9	121.5 (2)	C38—C39—C40	122.0 (2)
C11—C10—H10	119.3	C38—C39—H39	119.0
C9—C10—H10	119.3	C40—C39—H39	119.0
C10—C11—C6	120.6 (2)	C39—C40—C35	120.9 (2)
C10—C11—H11	119.7	C39—C40—H40	119.6
C6—C11—H11	119.7	C35—C40—H40	119.6
C9—C12—H12A	109.5	C38—C41—H41A	109.5
C9—C12—H12B	109.5	C38—C41—H41B	109.5
H12A—C12—H12B	109.5	H41A—C41—H41B	109.5
C9—C12—H12C	109.5	C38—C41—H41C	109.5
H12A—C12—H12C	109.5	H41A—C41—H41C	109.5
H12B—C12—H12C	109.5	H41B—C41—H41C	109.5
C2—N13—S14	117.79 (13)	C31—N42—S51	118.07 (13)
C2—N13—S22	117.50 (13)	C31—N42—S43	116.99 (13)
S14—N13—S22	123.86 (9)	S51—N42—S43	122.71 (9)
O1—S14—O2	120.91 (8)	O5—S43—O6	120.77 (9)
O1—S14—N13	104.42 (8)	O5—S43—N42	103.42 (8)
O2—S14—N13	105.63 (8)	O6—S43—N42	105.94 (9)
O1—S14—C15	108.55 (9)	O5—S43—C44	109.09 (10)
O2—S14—C15	109.88 (9)	O6—S43—C44	109.59 (9)
N13—S14—C15	106.39 (8)	N42—S43—C44	107.04 (9)
C20—C15—C16	120.91 (18)	C49—C44—C45	119.8 (2)
C20—C15—S14	118.98 (15)	C49—C44—S43	119.79 (17)
C16—C15—S14	120.08 (15)	C45—C44—S43	120.39 (17)
C17—C16—C15	118.92 (19)	C46—C45—C44	119.6 (2)
C17—C16—H16	120.5	C46—C45—H45	120.2
C15—C16—H16	120.5	C44—C45—H45	120.2
C16—C17—C18	121.72 (19)	C45—C46—C47	121.6 (2)
C16—C17—H17	119.1	C45—C46—H46	119.2
C18—C17—H17	119.1	C47—C46—H46	119.2
C17—C18—C19	118.33 (18)	C46—C47—C48	117.9 (2)
C17—C18—C21	121.4 (2)	C46—C47—C50	121.1 (2)
C19—C18—C21	120.3 (2)	C48—C47—C50	121.0 (2)
C20—C19—C18	121.4 (2)	C49—C48—C47	121.9 (2)
C20—C19—H19	119.3	C49—C48—H48	119.1
C18—C19—H19	119.3	C47—C48—H48	119.1
C19—C20—C15	118.67 (19)	C48—C49—C44	119.2 (2)
C19—C20—H20	120.7	C48—C49—H49	120.4
C15—C20—H20	120.7	C44—C49—H49	120.4
C18—C21—H21A	109.5	C47—C50—H50A	109.5
C18—C21—H21B	109.5	C47—C50—H50B	109.5
H21A—C21—H21B	109.5	H50A—C50—H50B	109.5
C18—C21—H21C	109.5	C47—C50—H50C	109.5
H21A—C21—H21C	109.5	H50A—C50—H50C	109.5
H21B—C21—H21C	109.5	H50B—C50—H50C	109.5
O4—S22—O3	121.39 (10)	O8—S51—O7	121.16 (10)
O4—S22—N13	107.88 (9)	O8—S51—N42	105.89 (9)
O3—S22—N13	103.99 (9)	O7—S51—N42	105.46 (8)
O4—S22—C23	109.14 (9)	O8—S51—C52	108.98 (9)
O3—S22—C23	108.52 (10)	O7—S51—C52	108.32 (10)
N13—S22—C23	104.60 (8)	N42—S51—C52	106.01 (8)
C24—C23—C28	120.01 (19)	C57—C52—C53	120.39 (19)
C24—C23—S22	120.34 (16)	C57—C52—S51	119.64 (15)
C28—C23—S22	119.63 (16)	C53—C52—S51	119.86 (16)
C25—C24—C23	120.1 (2)	C54—C53—C52	119.0 (2)
C25—C24—H24	120.0	C54—C53—H53	120.5
C23—C24—H24	120.0	C52—C53—H53	120.5
C24—C25—C26	121.3 (2)	C55—C54—C53	121.7 (2)
C24—C25—H25	119.4	C55—C54—H54	119.2
C26—C25—H25	119.4	C53—C54—H54	119.2
C27—C26—C25	117.8 (2)	C54—C55—C56	117.89 (19)
C27—C26—C29	121.4 (2)	C54—C55—C58	121.0 (2)
C25—C26—C29	120.8 (2)	C56—C55—C58	121.1 (2)
C28—C27—C26	121.8 (2)	C57—C56—C55	121.6 (2)
C28—C27—H27	119.1	C57—C56—H56	119.2
C26—C27—H27	119.1	C55—C56—H56	119.2
C27—C28—C23	119.0 (2)	C52—C57—C56	119.44 (19)
C27—C28—H28	120.5	C52—C57—H57	120.3
C23—C28—H28	120.5	C56—C57—H57	120.3
C26—C29—H29A	109.5	C55—C58—H58A	109.5
C26—C29—H29B	109.5	C55—C58—H58B	109.5
H29A—C29—H29B	109.5	H58A—C58—H58B	109.5
C26—C29—H29C	109.5	C55—C58—H58C	109.5
H29A—C29—H29C	109.5	H58A—C58—H58C	109.5
H29B—C29—H29C	109.5	H58B—C58—H58C	109.5
			
C5—S1—C2—N3	0.20 (16)	C34—S30—C31—N32	0.38 (16)
C5—S1—C2—N13	−178.73 (16)	C34—S30—C31—N42	179.08 (17)
N13—C2—N3—C4	179.31 (17)	N42—C31—N32—C33	−178.92 (17)
S1—C2—N3—C4	0.4 (2)	S30—C31—N32—C33	−0.2 (2)
C2—N3—C4—C5	−1.0 (2)	C31—N32—C33—C34	−0.1 (2)
C2—N3—C4—C6	178.96 (17)	C31—N32—C33—C35	−179.46 (16)
N3—C4—C5—S1	1.1 (2)	N32—C33—C34—S30	0.4 (2)
C6—C4—C5—S1	−178.80 (16)	C35—C33—C34—S30	179.67 (15)
C2—S1—C5—C4	−0.73 (16)	C31—S30—C34—C33	−0.41 (16)
C5—C4—C6—C11	−17.2 (3)	C34—C33—C35—C36	−179.7 (2)
N3—C4—C6—C11	162.9 (2)	N32—C33—C35—C36	−0.4 (3)
C5—C4—C6—C7	162.9 (2)	C34—C33—C35—C40	0.2 (3)
N3—C4—C6—C7	−17.0 (3)	N32—C33—C35—C40	179.45 (19)
C11—C6—C7—C8	−0.3 (3)	C40—C35—C36—C37	−0.6 (3)
C4—C6—C7—C8	179.6 (2)	C33—C35—C36—C37	179.3 (2)
C6—C7—C8—C9	−0.7 (4)	C35—C36—C37—C38	1.3 (4)
C7—C8—C9—C10	1.4 (3)	C36—C37—C38—C39	−1.2 (4)
C7—C8—C9—C12	−177.7 (2)	C36—C37—C38—C41	178.4 (2)
C8—C9—C10—C11	−1.1 (4)	C37—C38—C39—C40	0.3 (4)
C12—C9—C10—C11	178.0 (2)	C41—C38—C39—C40	−179.2 (2)
C9—C10—C11—C6	0.2 (4)	C38—C39—C40—C35	0.4 (4)
C7—C6—C11—C10	0.6 (3)	C36—C35—C40—C39	−0.3 (3)
C4—C6—C11—C10	−179.4 (2)	C33—C35—C40—C39	179.84 (19)
N3—C2—N13—S14	81.0 (2)	N32—C31—N42—S51	−81.3 (2)
S1—C2—N13—S14	−100.19 (16)	S30—C31—N42—S51	100.11 (16)
N3—C2—N13—S22	−88.9 (2)	N32—C31—N42—S43	82.2 (2)
S1—C2—N13—S22	89.99 (16)	S30—C31—N42—S43	−96.38 (16)
C2—N13—S14—O1	33.22 (15)	C31—N42—S43—O5	26.11 (16)
S22—N13—S14—O1	−157.66 (10)	S51—N42—S43—O5	−171.21 (10)
C2—N13—S14—O2	161.73 (13)	C31—N42—S43—O6	154.07 (14)
S22—N13—S14—O2	−29.15 (13)	S51—N42—S43—O6	−43.24 (13)
C2—N13—S14—C15	−81.50 (15)	C31—N42—S43—C44	−89.03 (15)
S22—N13—S14—C15	87.62 (12)	S51—N42—S43—C44	73.65 (13)
O1—S14—C15—C20	−29.21 (19)	O5—S43—C44—C49	−31.6 (2)
O2—S14—C15—C20	−163.45 (16)	O6—S43—C44—C49	−165.88 (17)
N13—S14—C15—C20	82.66 (18)	N42—S43—C44—C49	79.7 (2)
O1—S14—C15—C16	148.83 (16)	O5—S43—C44—C45	147.44 (19)
O2—S14—C15—C16	14.6 (2)	O6—S43—C44—C45	13.2 (2)
N13—S14—C15—C16	−99.30 (17)	N42—S43—C44—C45	−101.3 (2)
C20—C15—C16—C17	0.3 (3)	C49—C44—C45—C46	−0.6 (4)
S14—C15—C16—C17	−177.72 (15)	S43—C44—C45—C46	−179.7 (2)
C15—C16—C17—C18	−0.9 (3)	C44—C45—C46—C47	−0.6 (4)
C16—C17—C18—C19	0.8 (3)	C45—C46—C47—C48	1.3 (4)
C16—C17—C18—C21	−179.1 (2)	C45—C46—C47—C50	−178.0 (2)
C17—C18—C19—C20	−0.1 (3)	C46—C47—C48—C49	−0.8 (4)
C21—C18—C19—C20	179.8 (2)	C50—C47—C48—C49	178.5 (2)
C18—C19—C20—C15	−0.4 (3)	C47—C48—C49—C44	−0.4 (4)
C16—C15—C20—C19	0.3 (3)	C45—C44—C49—C48	1.1 (4)
S14—C15—C20—C19	178.36 (16)	S43—C44—C49—C48	−179.84 (18)
C2—N13—S22—O4	139.80 (14)	C31—N42—S51—O8	139.98 (14)
S14—N13—S22—O4	−29.35 (13)	S43—N42—S51—O8	−22.53 (13)
C2—N13—S22—O3	9.68 (16)	C31—N42—S51—O7	10.41 (16)
S14—N13—S22—O3	−159.47 (11)	S43—N42—S51—O7	−152.09 (11)
C2—N13—S22—C23	−104.10 (15)	C31—N42—S51—C52	−104.33 (15)
S14—N13—S22—C23	86.76 (12)	S43—N42—S51—C52	93.16 (12)
O4—S22—C23—C24	18.53 (19)	O8—S51—C52—C57	−145.10 (16)
O3—S22—C23—C24	152.78 (16)	O7—S51—C52—C57	−11.44 (19)
N13—S22—C23—C24	−96.69 (17)	N42—S51—C52—C57	101.34 (17)
O4—S22—C23—C28	−163.06 (17)	O8—S51—C52—C53	31.22 (19)
O3—S22—C23—C28	−28.81 (19)	O7—S51—C52—C53	164.87 (16)
N13—S22—C23—C28	81.72 (18)	N42—S51—C52—C53	−82.35 (17)
C28—C23—C24—C25	−0.1 (3)	C57—C52—C53—C54	0.0 (3)
S22—C23—C24—C25	178.32 (16)	S51—C52—C53—C54	−176.25 (16)
C23—C24—C25—C26	−1.5 (3)	C52—C53—C54—C55	0.1 (3)
C24—C25—C26—C27	2.2 (3)	C53—C54—C55—C56	0.1 (3)
C24—C25—C26—C29	−177.18 (19)	C53—C54—C55—C58	179.68 (19)
C25—C26—C27—C28	−1.3 (3)	C54—C55—C56—C57	−0.4 (3)
C29—C26—C27—C28	178.0 (2)	C58—C55—C56—C57	−179.97 (19)
C26—C27—C28—C23	−0.2 (3)	C53—C52—C57—C56	−0.3 (3)
C24—C23—C28—C27	0.9 (3)	S51—C52—C57—C56	175.97 (15)
S22—C23—C28—C27	−177.50 (16)	C55—C56—C57—C52	0.5 (3)

### Hydrogen-bond geometry (Å, º)

**Table:**

*D*—H···*A*	*D*—H	H···*A*	*D*···*A*	*D*—H···*A*
C29—H29*B*···O3^i^	0.96	2.54	3.148 (3)	122
C50—H50*C*···O5^ii^	0.96	2.47	3.397 (3)	162

Symmetry codes: (i) −*x*+1, −*y*+2, −*z*+1; (ii) −*x*+1, −*y*+2, −*z*.
